# Cell-Biological Requirements for the Generation of Dentate Gyrus Granule Neurons

**DOI:** 10.3389/fncel.2018.00402

**Published:** 2018-11-12

**Authors:** Maryam Hatami, Sabine Conrad, Pooyan Naghsh, Gonzalo Alvarez-Bolado, Thomas Skutella

**Affiliations:** ^1^Institute for Anatomy and Cell Biology, Heidelberg University, Heidelberg, Germany; ^2^Independent Researcher, Tübingen, Germany; ^3^Department of Biochemistry and Molecular Biology, University of Calgary, Calgary, AB, Canada

**Keywords:** dentate gyrus, granule cells, induced pluripotent stem cells (iPSC), *in vitro*, requirements

## Abstract

The dentate gyrus (DG) receives highly processed information from the associative cortices functionally integrated in the trisynaptic hippocampal circuit, which contributes to the formation of new episodic memories and the spontaneous exploration of novel environments. Remarkably, the DG is the only brain region currently known to have high rates of neurogenesis in adults (Andersen et al., 1966, 1971). The DG is involved in several neurodegenerative disorders, including clinical dementia, schizophrenia, depression, bipolar disorder and temporal lobe epilepsy. The principal neurons of the DG are the granule cells. DG granule cells generated in culture would be an ideal model to investigate their normal development and the causes of the pathologies in which they are involved and as well as possible therapies. Essential to establish such *in vitro* models is the precise definition of the most important cell-biological requirements for the differentiation of DG granule cells. This requires a deeper understanding of the precise molecular and functional attributes of the DG granule cells *in vivo* as well as the DG cells derived *in vitro*. In this review we outline the neuroanatomical, molecular and cell-biological components of the granule cell differentiation pathway, including some growth- and transcription factors essential for their development. We summarize the functional characteristics of DG granule neurons, including the electrophysiological features of immature and mature granule cells and the axonal pathfinding characteristics of DG neurons. Additionally, we discuss landmark studies on the generation of dorsal telencephalic precursors from pluripotent stem cells (PSCs) as well as DG neuron differentiation in culture. Finally, we provide an outlook and comment critical aspects.

## Development of the Dentate Gyrus (DG) at the Histological Level (Figure 1)

Before thinking of producing dentate gyrus (DG) granule cells (or the entire DG itself) *in vitro*, it is convenient to review the formation of the DG in the mouse brain at the histological level. Key structures for DG development are: (1) the cortical hem (CH) and the anti-hem; (2) the radial glia; (3) the Cajal-Retzius cells (CR); and (4) the surrounding meninges. DG development is regulated by growth factor gradients which in turn activate transcription factor expression in the corresponding precursor cells and in the differentiating neurons. The DG originates in three proliferative matrices and the cellular events leading to its formation can be best described as subdivided into three developmental stages.

**Figure 1:**
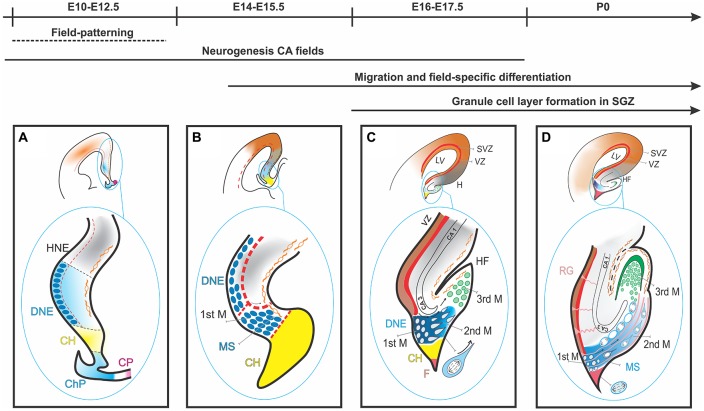
Dentate gyrus (DG) development in the mouse from E10 to postnatal stage. **(A)** The migration and differentiation of granule cells is controlled by the CH, a glial scaffold formed by radial glial cells (RGCs) and CR lining the HF. At E10–E12.5 DG precursor cells (small dark blue) start to develop in the DNE, adjacent to CH (light blue). **(B)** By E14–E15.5 the first migration of DG precursors is initiated. **(C)** By E17.5 DG precursors and granule cells have begun to mix and form the 2ndM and 3ndM. **(D)** By P0 condensing of granule cell layers (GCLs) in subgranular zone. HNE, Hippocampus Neuroepithelium; DNE, Dentate Neuroepithelium; CH, Cortical Hem; ChP, Choroid Plexus; CP, Coroid Plaque; 1stM, First Matrix; MS, Migratory Stream; CR, Cajal Retzius; 2ndM, Second Matrix; 3ndM, Third Matrix; HF, hippocampal fissure; F, Fimbria.

### First Stage

DG development begins with the expansion of the undifferentiated neuroepithelium sheet (ventricular zone, VZ) of the early cortical primordium. During this phase, signal morphogens are secreted from two discrete telencephalic regions: the CH, a long and narrow region caudal and adjacent to the presumptive hippocampal neuroepithelium, and the more rostrally located anti-hem. The CH is considered one of the organizers (or patterning centers Rubenstein and Beachy, 1998; Grove and Fukuchi-Shimogori, 2003). The morphogens provide positional cues and regulate the regional proliferation and architecture in the developing cortex. The cortical neuroepithelium responds to these signals by generating a cortical proto-map (Grove and Fukuchi-Shimogori, 2003) through region-specific neurogenesis and gliogenesis. The first subset of proliferative neuroepithelial cells of the DG (i.e., the first proliferative matrix or the DG presumptive neuroepithelium) is regionalized as part of the medial pallium VZ.

### Second Stage

DG progenitors go on dividing by mitosis as they leave the VZ and migrate towards the hippocampal fissure (HF); these migrating precursors constitute the secondary proliferative matrix. This is a unique phenomenon: a migrating population of mitotically active progenitors at various differentiation stages. The migration is guided by a radial glial scaffold, which originates from the fimbrial region and leads to the pial side of the cortex and the HF. Additionally, a special kind of neurons, the CR cells, originating in the CH and migrating towards the HF, provide factors essential for DG precursor migration (Rickmann et al., 1987; Del Río et al., 1997). CR cells express reelin (*Reln*), which encodes a protein essential for the migration of neurons along radial glia. The formation of the radial glial scaffold is triggered by a cascade of interactions involving *Reln*, Disabled1 (*Dab*) and Integrin beta1 (*Itbg1*; Förster et al., 2002). In mice deficient in *Reln*, the cell-dense DG granule layer, precisely demarcated from the hilus (Förster et al., 2002), fails to form, and the granule cells are misplaced in the hilus area (Förster et al., 2002).

### Third Stage

Upon arrival to the HF, the DG migrating progenitors (secondary proliferative matrix) accumulate in this region and form the tertiary proliferative matrix. Granule cells originate in all three matrices. CR cells surrounding the HF and the pial surface define the granule cell layer (GCL)’s well-known V-shaped progenitor and granule cells become gradually limited to the tertiary matrix. In the early postnatal phase, the subgranular zone (i.e., the presumptive adult hippocampal neurogenesis site) develops from the tertiary matrix (Bayer, 1980a, b; Altman and Bayer, 1990; Pleasure et al., 2000; Brunne et al., 2010; Khalaf-Nazzal and Francis, 2013; Sugiyama et al., 2013).

Let us mention here briefly the development of axonal connections, a complex subject in itself, which has been often reviewed in this context (see for instance Skutella and Nitsch, 2001). During development, the formation of hippocampal connections is directed by a complex guidance signaling network. The developing hippocampus originates outgrowing axons which navigate using long-range cues including secreted class3 Semaphorins, Netrin1 and Slit proteins. Layer-specific positional information is communicated by regional molecules in the membrane or anchored in substrates like ligands of the Ephrin A subclass. In the adult mouse DG, the silencing of semaphorin receptors neuropilins1 or 2 in neural progenitors *in vivo* causes newly differentiated neurons with shorter dendrites and simpler branching (Xu C. J. et al., 2015).

## Functional Integration of Newborn DG Granule Cells

Although in the mouse the first DG granule cells are generated during the final phase of embryogenesis, most granule cell neurogenesis occurs within the first two postnatal weeks. After that, the rate of granule cell production decreases significantly (about 90% less neurons are generated in rats and humans of medium age in comparison to young animals; Schlessinger et al., 1975; McDonald and Wojtowicz, 2005; Knoth et al., 2010; Kempermann, 2011; Lopez-Rojas and Kreutz, 2016). This reduced neurogenesis correlates with the decline in cognitive capabilities that is typical of aging (Drapeau and Nora Abrous, 2008; Seib and Martin-Villalba, 2015), and it could be the cause of certain deficits in pattern separation also associated with the aging process (Sahay et al., 2011; Yassa et al., 2011; Holden and Gilbert, 2012).

The functional (electrophysiological) maturation of hippocampal neurons is probably regulated by a genomic network mostly independent from external stimuli; this would explain the fact that the sequence of events leading to the functional (electrophysiological) differentiation of hippocampal neurons is the same for neurons generated in embryonic and early postnatal brains and for neurons generated in the adult (Espósito M. S. et al., 2005). Accurate descriptions of the physiology of postnatally generated DG granule cells are available (adult neurogenesis in the DG and its functional implications have been reviewed in detail recently (Christian et al., 2014; Yu et al., 2014b; Abrous and Wojtowicz, 2015; Opendak and Gould, 2015). In the adult, DG granule cells originate from neuronal stem cells from the subgranular zone. During the 1st week of their generation, and right after commitment to the neuronal lineage, the early neuroblasts drift towards the inner granular cell layer and send out the first cellular processes. However, these neuroblasts are not fully involved in the trisynaptic network and they show electrical activity when excited by ambient γ-aminobutyric acid (GABA), not glutamate (Espósito M. S. et al., 2005). During the 2nd week, fast growth of neurites and synaptogenesis are characteristic, as the essential integration of the DG into the synaptic network takes place. Over 50% of cells generated from adults do not integrate and undergo apoptosis (Gould et al., 1999; Dayer et al., 2003; Sierra et al., 2010). GABA triggers the first functional synaptic inputs in young granule cells. During the 3rd week, the new DG granule cells start to receive glutamatergic axons from the entorhinal cortex and to build the corresponding postsynaptic contacts in their dendrites (Espósito M. S. et al., 2005; Overstreet Wadiche et al., 2005). Dendritic spines start to appear in granule cells from week 2 on, and their number constantly increases until the 8th week, when it reaches its maximum. Afterwards, spines continue to mature until week 18. Spine motility undergoes dynamic changes, which are maximal in the 4th to 8th weeks and diminish afterwards (Zhao et al., 2006).

Early during the 2nd week, the axons of the granule cells mature and form synaptic contacts with CA3 postsynaptic targets; however, the contacts are stable only from the 4th week on (Zhao et al., 2006; Gu et al., 2012). Eight weeks after their generation, granule cells have reached their final anatomical destination and show mature function. During this phase they can barely be discerned from granule cells generated during embryonic and early postnatal development (Laplagne et al., 2006; Ge et al., 2007; Mongiat et al., 2009).

The functional integration of DG granule cells is also possible in culture. It has been reported that, after 3 weeks of differentiation, cultures of immature DG granule neurons on hippocampal astrocytes show functional neural networks (Yu et al., 2014a). Somatic intracellular Ca^2+^ dynamics obtained from selected regions of these cultures reflects neuronal activity patterns of hippocampal granule cells and can be used as a proxy of spontaneous activity and functional connectivity. Furthermore, transplantation of pre-patterned hippocampal NPCs into the DG of perinatal mice gives rise to functional neurons in the GCL that are properly integrated into the hippocampal neural circuitry (Yu et al., 2014a).

## Morphogenetic Proteins and Growth Factors Essential for the Generation of DG Granule Cells (Figure 2)

**Figure 2:**
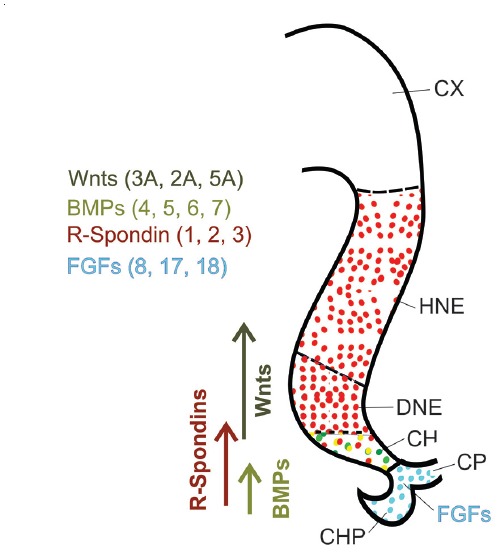
Major secreted proteins and growth factors in hippocampal development at E11.5. WNT and bone morphogenetic protein (BMP) ligands are secreted from the CH, while the ChP plexus secretes fibroblast growth factors (FGFs). R-Spondins are secreted proteins expressed not only in the CH but also widely in the hippocampal neuroepithelium.

**Figure 3:**
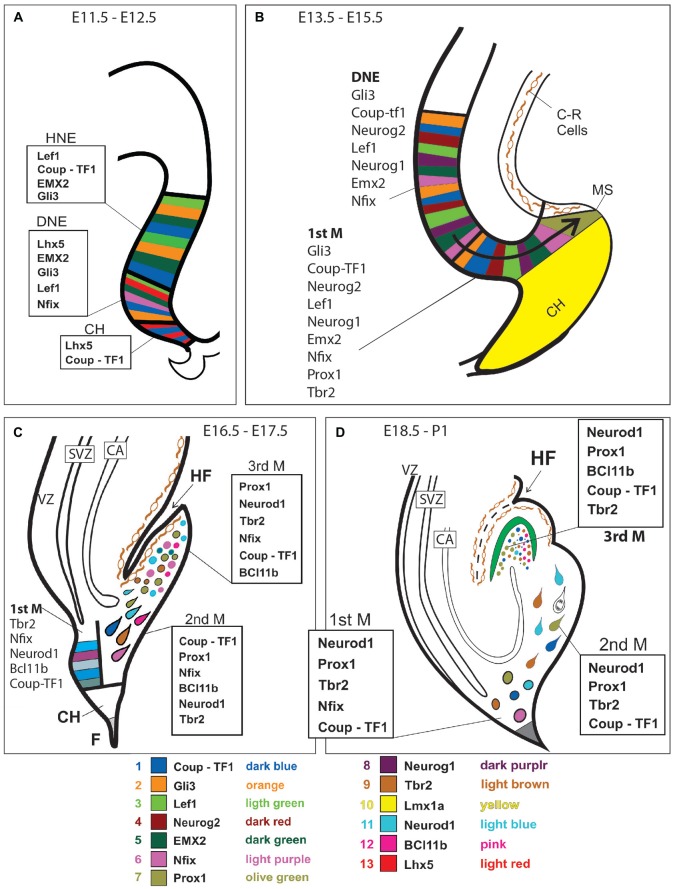
Transcription factor expression in DG development. Expression of the most important transcription factors is represented at four different developmental ages (as indicated) and superimposed to diagrams of the corresponding histological events. Possible cellular colocalization of markers has not been represented. See text for details. Gene expression is depicted at E11.5-E12.5 **(A)**; at E13.5-E15.5 **(B)**; at E16.5-E17.5 **(C)**, and at E18.5-P1 **(D)**.

**Figure 4:**
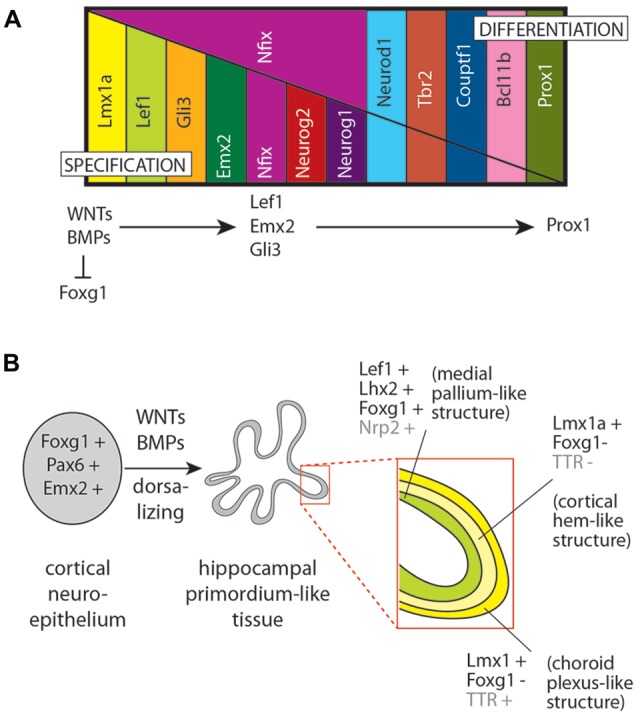
A hierarchy of factors involved in DG development *in vivo* and in culture. **(A)** Expression of a series of transcription factors during specification and then differentiation of the DG. Although a strict hierarchy of regulators serially activating each other is not yet available, the general rule that WNT and BMP ligands are requested to repress cortical specification (as indicated by forkhead box G1 (*Foxg1*) expression) and to confer DG specification (as indicated by *Lef1* and *Emx2* expression), which in turn are requested for downstream differentiation of DG granule cells (specifically expressing prospero homeobox 1 (*Prox1*)). **(B)** Application of the principle shown in **(A)** to the development of hippocampal organoids in culture. Embryonic cortical neuroepithelium (typically expressing *Foxg1*, *Emx2* and paired box 6 (*Pax6*); left side) is treated with WNT and BMP ligands to give rise to a hippocampal primordium-like tissue (center) with layers (right side insert) showing transcription factor gene expression specific for the medial pallium, the CH and the ChP (as indicated; based on data from Sakaguchi et al., 2015; the genes *Nrp2* (neuropilin 2) and *TTR* (transthyretin) are not transcription factors but they show region-specific expression useful to identify anatomical structures).

In Sections “Morphogenetic Proteins and Growth Factors Essential for the Generation of DG Granule Cells (Figure 2),” to “Other Proteins Necessary for the Maintenance of DG Granule Cells in Postnatal and Adult Stages: Tlx and Ccnd2,” we will outline key components of the molecular framework underlying the visible developmental stages that we just described. First, we will focus on essential secreted morphogenetic proteins indispensable for DG granule cell development. Under the influence of the signaling cascades activated by these proteins, early precursor cells will express combinations of transcription factors that we will examine in Sections “Some Transcription Factors Essential for DG Granule Cell Development (Figures 3, 4)” and “Some Transcription Factors Essential for DG Granule Cell Migration (2).”

### Essential Morphogenetic Proteins in DG Development

The CH secretes bone morphogenetic proteins (BMPs) and WNT protein ligands (as well as growth and differentiation factors (GDFs); see Sections “Fibroblast Growth Factors (FGFs)” and “Cytokine SDF1 Secreted by Cajal-Retzius cells”). The anti-hem secretes fibroblast growth factor 7 (FGF7), epidermal growth factor (EGF) and sonic hedgehog (SHH), as well as secreted frizzled-related protein 2 (SFRP2; an inhibitor of WNT signaling; Tole et al., 1997; Grove and Tole, 1999). Furthermore, the meninges surrounding the developing DG secrete BMP7 (Choe et al., 2013). In addition, the CH expresses *R-spondins* (*Rspo*), encoding secreted modulators of WNT signaling, whose functions in the DG have not been analyzed yet.

#### WNT Ligands From the Cortical Hem

In the mouse, hippocampus development begins around embryonic day 14 in reaction to BMP and WNT ligands secreted by the CH (Grove et al., 1998; Mangale et al., 2008), and disruption of this structure results in abnormal hippocampal development (Yoshida et al., 2006). The WNT ligands secreted by the CH (WNT2a, -2b, -3a and -5a), are major players in the proliferation and organization of hippocampal neural precursors (Furuta et al., 1997; Galceran et al., 1999; Lee et al., 2000; Caronia et al., 2010). Key among them is *Wnt3a*, whose expression starts very early and is restricted to the CH (Grove et al., 1998; Lee et al., 2000). In mutant mice deficient in *Wnt3a*, the DG is absent at the medial border of the hippocampus formation, while the CA3 and CA1 regions and the subiculum are absent rostrally and very decreased in size caudally. Deficiency in low density lipoprotein receptor-related protein 6 (*Lrp6*, the WNT receptor) causes severe hippocampal abnormalities (Yoshida et al., 2006). Additionally, the expression of multiple WNT modulators in the CH strongly suggests a requirement for tight regulation of WNT/β-catenin signaling during hippocampal development (see below, Section “Gli3”). An essential transcription factor downstream Wnt signaling in this region is lymphoid enhancer binding factor 1 (LEF1; see below, Section “Lef1”).

#### BMP Ligands From the Cortical Hem

Various BMPs, including BMP4, -5, -6 and -7, are secreted by the telencephalic roof plate in an early embryonic phase, and by the CH in a later phase (Furuta et al., 1997; Grove et al., 1998; Hebert et al., 2002). A complete lack of BMP signaling leads to missing telencephalic medio-dorsal structures, including choroid plexus (ChP) and CH, which in turn results in complete lack of hippocampal development (Cheng et al., 2006; Fernandes et al., 2007). Upon formation of the CH, BMPs seem to be no longer necessary for determining the early hippocampal cell properties (Hebert et al., 2002). Due to the differential functions of receptors BMPR-1a and BMPR-1b, BMP ligands have different effects on neural precursors: while proliferation of the embryonic telencephalon is promoted by BMPR-1a, cell cycle arrest and differentiation are supported by BMPR-1b (Panchision et al., 2001).

In addition, some properties of BMP ligands in the adult DG are of interest for researchers testing protocols for the *in vitro* differentiation of DG granule cells. BMPs play a crucial role in maintaining neural stem cell (NSC) quiescence (Mira et al., 2010). The morphogen proteins NOGGIN and CHORDIN antagonize BMPs in the adult hippocampal proliferation niche (Scott et al., 2000; Fan et al., 2003; Bonaguidi et al., 2005, 2008). Depletion of the BMPR-1a receptor subunit results in overproduction of neurons (to the expense of proliferating NSCs) by adult NSCs and therefore reduces their population (Mira et al., 2010). BMPs can also trigger NSCs quiescence in culture (Mira et al., 2010; Sun et al., 2011; Martynoga et al., 2013) and they can support astrocytic gene expression *in vitro*, which again allows the induction of a selection of astroglial features of adult NSCs (Gross et al., 1996; Sun et al., 2011). The fact that BMPs are essential for the quiescence of NSCs as well as for the differentiation and maturation of granule cells (Bond et al., 2014), can be explained by the differential expression of BMPR-1 receptors. In the adult DG, NSCs express BMPR-1a, which is then downregulated in intermediate precursor cells (IPCs). Contrarily, neurons express BMPR-1b (Lim et al., 2000; Mira et al., 2010).

Finally, BMP acts through the activin receptor type 1 (ACVR1) which in turn induces *Lef1* expression (Choe et al., 2013). *Lef1* is a key transcription factor gene activated not only by WNT ligands but also by BMPs (Armenteros et al., 2018; see below, Section “Lef1”).

#### Sonic Hedgehog (SHH)

Secreted morphogen protein SHH, in combination with WNT signaling, is involved in later developmental stages of the DG. It plays an important role in expanding the granule neural progenitor population during perinatal development (Machold et al., 2003). SHH and its receptor patched 1 (PTCH1) are expressed during early postnatal phases in the hilar DG regions, CA3, and along fimbria fibers (Lai et al., 2003; Machold et al., 2003). Ablation of *Smoothened* (*Smo*), encoding a component of the SHH receptor complex, leads to abnormalities in regions of postnatal neurogenesis, e.g., a reduced amount of granule DG cells compared to other hippocampal regions (Machold et al., 2003; Han et al., 2008). DG cell proliferation can also be reduced by pharmacological inhibition of SHH signaling through cyclopamine (Lai et al., 2003). In contrast, increased SHH signaling *in vitro* causes higher DG cell proliferation, which confirms that SHH has mitogenic effects *in vivo* (Lai et al., 2003; Machold et al., 2003; Han et al., 2008).

### Fibroblast Growth Factors (FGFs)

Several members of the FGFs family of secreted proteins with growth and differentiation functions are expressed in the early ChP and elicit specific gene expression in the adjacent primordium of the hippocampus, including the DG (Zimmer et al., 2010). The gene for Fgf receptor 1 (*Fgfr1*), necessary for FGFs to exert their effects on the target cells is expressed in the entire hippocampus (CA and DG), from the neuronal precursors of the neuroepithelium to the postmitotic pyramidal cells and DG granule cells; mutants deficient in *Fgfr1* have 30%–50% less granule cells in the DG (Ohkubo et al., 2004).

### Cytokine SDF1 Secreted by Cajal-Retzius Cells

We have already mentioned reelin (RELN), a protein secreted by the Cajal-Retzius neurons (see above, Section “Development of the DG at the Histological Level (Figure 1)”). The chemokine Stromal cell-derived factor 1 (SDF1, CXCL12) is a chemoattractive protein also secreted by CR cells to help DG granule precursors migrate from the neuroepithelium to the DG granule layer (Bagri et al., 2002). In mouse mutants deficient in chemokine receptor type 4 (CXCR4), the DG and the migration path contain less proliferating cells, differentiation is untimely and they have a smaller DG lacking its well-known V-shape (Lu et al., 2002). This indicates that CR cells do not directly determine granule cell development, but DG formation (cytoarchitecture).

### Summary

The interplay between WNT and BMP ligands and SHH establishes and sustains the DG stem cell niche (Galceran et al., 2000; Chenn and Walsh, 2002; Machold et al., 2003; Zhou et al., 2004; Lie et al., 2005; Machon et al., 2007; Favaro et al., 2009; Caronia et al., 2010; Mira et al., 2010; Munji et al., 2011). FGFs stimulate proliferation and are essential for both Ammon’s horn (CA) and DG to reach the appropriate number of cells (Ohkubo et al., 2004). Therefore, for the early generation of DG granule cells in culture, it will be important to use WNT and BMP ligands for specification as well as FGFs to stimulate proliferation.

## Some Transcription Factors Essential for DG Granule Cell Development (Figures 3, 4)

The secreted proteins reviewed above elicit expression of transcription factor genes which further the proliferation, migration and differentiation of granule cell precursors. When trying to produce DG granule cell *in vitro*, the expression of these transcription factors can be used to determine if addition of BMPs, WNTs, et cetera to the culture has effected the desired results. Alternatively, forced expression of combinations of these transcription factors can be attempted in culture in order to steer the differentiation of progenitor cells.

A myriad of transcription factors are involved in the development of such a complex structure as the DG. Here we will review some particularly important, especially if their relevance to the production of DG neurons in culture has been shown. This selection has to be necessarily limited to a few really indispensable as markers of key stages in DG granule cell development.

### Lef1

Among the nuclear mediators of WNT signaling are *Lef1* and other genes of the TCF/LEF family of transcription factors (Galceran et al., 2000). *Lef1* is highly expressed in the neuronal precursors of the DG and, less intensely in those of the adjacent (CA) region (Allen Brain Atlas; Lein et al., 2007). *Lef1*-deficient mouse embryos specifically lack any DG granule cells (Galceran et al., 2000). The entire hippocampal area including the CA fields is absent in mouse embryos homozygous for a *Lef1-lacZ* fusion gene encoding a protein which not only lacks DNA binding domain but impairs transcriptional activation by other LEF/TCF proteins. Consequently, the generation of DG granule cells is controlled by LEF1, which also regulates hippocampal regional development in combination with other LEF/TCF proteins.

### Gli3

We have mentioned above SHH (Section “Morphogenetic Proteins and Growth Factors Essential for the Generation of DG Granule Cells (Figure 2)”), a secreted morphogen protein that exerts its effects through the GLI-Kruppel family of conserved zinc finger-transcriptional regulators. Dorsal telencephalon development is controlled at an early stage by Gli-Kruppel family member GLI3 (Theil et al., 1999). The brain of the extra-toesJ (XtJ/XtJ) mutant mouse, which carries a deletion in *Gli3*, lacks entirely CA, DG and plexus choroideus of the lateral ventricle. The targets of GLI3 are WNT ligand genes including *Wnt3a*, *Wnt8b* and *Wnt9a* as well as several modulators of WNT signaling, like the WNT signaling pathway inhibitor Dickkopf 2 (*Dkk2*), APC down-regulated 1 (*Apcdd1*), *Rspo1-3* and *Sfrp1* (Hasenpusch-Theil et al., 2012). Except for *Sfrp1*, the expression of these genes is restricted to the CH (*Rspo3* shows additional weak expression in hippocampal progenitors adjacent to the CH and in CR cells).

### Emx2

Transcription factor gene *Emx2* is expressed within the diencephalon roof with a caudo-rostral gradient (Simeone et al., 1992). *Emx2* is essential for the development of the DG, and mouse embryos deficient in *Emx2* completely lack a DG (Pellegrini et al., 1996; Oldekamp et al., 2004a). A defect in the cortical positional information-signal cascade, which is transduced by FGF8 and probably WNT proteins, might be the cause of the phenotype (Muzio et al., 2002; Fukuchi-Shimogori and Grove, 2001; Ligon et al., 2003; Shimogori et al., 2004). CR cells in the marginal zone of the DG, which also express *Emx2*, are reduced in number in *Emx2*-deficient mouse brains (Mallamaci et al., 1998, 2000). The *Emx2*-expressing cells of the DG neuroepithelium coexpress Neurogenin1 (*Neurog1*), neurogenin 2 (*Neurog2*) and T-box TF2 (*Tbr2*; see below).

### Neurog2

*Neurog2* is a bHLH transcription factor gene with proneural function expressed in the embryonic neuroepithelium. It promotes the neuronal fate in multipotent stem cells. *Neurog2* activates the expression of several neuronal differentiation genes, e.g., the NeuroD family of transcription factors (Seo et al., 2007; Wilkinson et al., 2013), and it plays a crucial role in the differentiation of glutamatergic neurons (Schuurmans et al., 2004; Berninger et al., 2007; Wilkinson et al., 2013). During DG formation, all proliferative matrix precursor cells express *Neurog2* (Pleasure et al., 2000; Galichet et al., 2008). *Neurog2* null mutants show a severely atrophic DG at birth due to defects in DG proliferation and differentiation (Galichet et al., 2008). Although *Neurog2*-deficient progenitor cells expressed *Ascl1* during DG morphogenesis, this gene could not compensate the lack of *Neurog2* in the DG (although it can in the rest of the telencephalon; Nieto et al., 2001; Galichet et al., 2008). The glial scaffold of the *Neurog2* mutant DG is malformed, causing an interrupted migration of progenitors (Galichet et al., 2008). Since the atrophic DG and disorganized glial scaffold appear also in *Wnt* mutant embryos, it is possible that *Neurog2* acts downstream WNT signaling during DG formation (Hirabayashi et al., 2004; Zhou et al., 2004; Galichet et al., 2008).

### Neurod1

The transcription factor gene Neuronal differentiation 1 (*Neurod1*) is a bHLH proneural gene expressed with a dorso-ventral gradient in the cortical plate and tertiary matrix of the DG. The DG and cerebellum are completely missing in *Neurod1* conditional knockouts (Miyata et al., 1999) because this gene is required for the survival of DG granule cells (Miyata et al., 1999; Liu et al., 2000; Schwab et al., 2000). *Neurod1* overexpression causes premature neuronal maturation (Roybon et al., 2009; Boutin et al., 2010).

### Prox1

Prospero homeobox 1 (*Prox1*) is a transcription factor gene expressed by the migration stream of newly generated granule cell precursors in the secondary matrix and also by mature granule cells in the tertiary matrix of the emerging DG (Oliver et al., 1993; Oldekamp et al., 2004b; Li et al., 2009). It is therefore frequently utilized as a DG granule neuron lineage marker and plays an essential role in proliferating neuronal progenitors and granule cells in DG development (Rubin and Kessaris, 2013). Studying *Prox1* null mutant mice made evident that *Prox1* plays an essential role in proliferating neuronal progenitors and differentiation of granule cells in DG development (Lavado et al., 2010). Remarkably, postmitotic DG granule neurons deficient in *Prox1* trans-differentiate into CA3 pyramidal neurons (Iwano et al., 2012).

### Nfix

The transcription factor nuclear factor I/X (*Nfix*) is necessary for NSCs to find their specific location in the emerging DG (Martynoga et al., 2013). *Nfix* is already intensely expressed in the DG neuroepithelium on embryonic day 14, and *Nfix* null mutant mice display severe defects in DG formation because of impaired differentiation of neurons and glial cells. In *Nfix* mutants, *Prox1*-expressing granule neurons are reduced in number, their glial scaffold is disordered and DG morphogenesis is inappropriate (Martynoga et al., 2013; Heng et al., 2014). *Nfix* regulates a large number of genes, many of which influence cell motility and adhesion as well as extracellular matrix generation (Martynoga et al., 2013).

### Bcl11b

*Bcl11b* (*Ctip2*) is a transcription factor gene expressed in DG granule cells. Its function affects two essential developmental processes, i.e., the proliferation and the differentiation of granule cells. Since *Bcl11b* is expressed in postmitotic DG granule neurons, its influence on the proliferation compartment is probably effected through secretion of a factor acting on dividing cells of the proliferation matrix (positive feedback). A direct transcriptional target of BCL11B, the cell-cell adhesion protein desmoplakin (DSP), is essential for the proper differentiation of DG granule cells (Simon et al., 2012).

## Some Transcription Factors Essential for DG Granule Cell Migration (Figure 2)

### Tbr2

Same as *Neurog2*, *Tbr2* is expressed in all three DG proliferative matrices by proliferating progenitor cells (Hodge et al., 2012), and its deletion inhibits the production of IPCs and granule neurons and enhances the proliferation of stem cells. This suggests that *Tbr2* plays a role in the reconfiguration of stem cells into late differentiating IPCs. *Tbr2* could accomplish this function by direct downregulation of *Sox2*, a stem cell transcription factor (Hodge et al., 2012). *Tbr2* is also expressed by CR cells originating from the CH; in *Tbr2* mutant mice, these neurons proliferate deficiently causing abnormal DG morphogenesis (Hodge et al., 2013).

### Lhx5

In LIM homeobox protein 5 (*Lhx5*) knockout mice, DG precursor cell migration is disturbed so that they accumulate in the neuroepithelium (VZ). This results in the absence of DG granule cells (Zhao et al., 1999). In these mutants, the fimbria and hippocampal commissures do not develop either.

### Lmx1a

*Lmx1a* is a transcription factor gene expressed in the CH and essential for the specification of its derivatives (Chizhikov et al., 2010). In particular, *Lmx1a* stimulates the proliferation of CR cells, probably by repressing *Lhx2* (Chizhikov et al., 2010). As mentioned above, Cajal-Retzius neurons secrete RELN, which in turn is essential for the DG precursors to migrate along radial glial fibers. Mouse brains deficient in *Lmx1a* (the classical *dreher* mutant) show a DG GCL partially absent, cytoarchitectonically disorganized (Sekiguchi et al., 1992) and with gross alterations in the arrangement of axonal afferents (Sekiguchi et al., 1996).

### Couptf1 (Nr2f1)

*Couptf1* is a transcription factor gene expressed at every stage of DG granule cell development, i.e., dentate neuroepithelium, proliferating precursors and differentiated granule cells (Parisot et al., 2017). *Couptf1* is a direct activator of the migration-related cytokine *Cxcr4* and is essential for the formation of the radial glial fiber scaffold that supports and guides the migration of the DG precursors. Since radial glial cells (RGCs) are also responsible for the proliferation of DG precursors in the first matrix (1stM; neuroepithelium), *Couptf1* is positioned at the crossroads between precursor proliferation and migration, two cellular phenomena tightly linked in the DG (Parisot et al., 2017).

## Other Proteins Necessary for the Maintenance of DG Granule Cells in Postnatal and Adult Stages: Tlx and Ccnd2

Interesting at postnatal and adult ages are two other factors which, although not relevant during development, are required for the maintenance of the DG granule cells and worth mentioning briefly here: *Tlx* (a transcriptor factor) and *Ccnd2* (a cyclin). While *Tlx* is dispensable for DG development, it is essential for adult neurogenesis from the subgranular zone (DG) and VZ-SVZ (fated for the olfactory bulb; Shi et al., 2004; Zhang et al., 2008; Niu et al., 2011; Murai et al., 2014). In the DG of wild-type mice, *Tlx* overexpression induces neurogenesis and enhances memory and learning (Murai et al., 2014), suggesting that, in NSCs, *Tlx* induces the transition from quiescence to activation. In addition, *Tlx* activates *Ascl1* for induction of neuronal lineage commitment, activates WNT signal induction for NSC proliferation and regulates astrogenesis by BMP signal downregulation (Shi et al., 2004; Elmi et al., 2010; Qu et al., 2010; Qin et al., 2014).* Ccnd2* is also dispensable for hippocampal development but essential for late postnatal and adult neurogenesis in the hippocampal niche (Kowalczyk et al., 2004; Shi et al., 2004; Ansorg et al., 2012). CCND2 directs the cell cycle transition between the G1 and S mitotic phases and is a key component of the cell cycle mechanism, together with CCND1 and CCND3 and the cyclin-dependent kinases (CDKs; Sherr et al., 1994; Ekholm and Reed, 2000).

## Programmed Differentiation of Telencephalic Precursors and DG Neurons From ESCs

The generation of DG granule cells *in vitro*, from mouse and from human tissue, has been attempted using different strategies that mimic essential cellular and molecular events of telencephalic differentiation and hippocampus development (Watanabe et al., 2005; Yu et al., 2014a; Sakaguchi et al., 2015).

The idea underlying the approaches taken by the laboratories of Sasai and Gage was to imitate normal development as close as possible (signaling pathways, growth factors, etc.,) while keeping specific cell culture conditions. Embryoid bodies (EBs) and single culture systems were used as starting point, and sometimes pre-differentiated cells were transplanted to brains *in vivo* for the ultimate differentiation into recognizable cell types, including DG granule cells. Both the laboratories of Sasai and Gage could generate ESC/iPSC-derived hippocampal/DG-like neurons by 3D-culturing techniques and by applying some key growth factor modulating hippocampal development. Comparing both cultivation methods, Sakaguchi et al. (2015) focused more on the early hippocampal developmental stage. The application of the hippocampal differentiation protocols to hiPSCs from patients with hippocampal pathologies could provide new insights into neuropathology and the discovery of new targets for pharmaceutics (Yu et al., 2014b).

By using an optimized serum-free suspension culture with addition of the WNT antagonist DKK1, FOXG1-positive telencephalic cells can be generated; these can be made to express typical dorsal telencephalic markers paired box 6 (PAX6) and EMX1 by the subsequent administration of WNT3a.

One protocol based on EBs formed from hESC-derived dorsomedial telencephalic cells cultured in the presence of a combination of growth factors and BMP and WNT agonists was successful in generating two hippocampal neuronal types. One of these neuronal populations expressed the pan-hippocampal marker gene *Zbtb20* as well as *Prox1* (DG granule cell marker), while the other expressed *Zbtb20* in combination with glutamate ionotropic receptor kainate type subunit 4 (*GRIK4*, also known as *KA1*), a marker of pyramidal cells of CA. However, these cells showed immature firing patterns and did not achieve the reconstruction of the primary hippocampal neuronal circuitry: specific CA1 pyramidal neurons could not be generated, and the formation of the DG or regionally specific hippocampal tissues could not be recapitulated (Sakaguchi et al., 2015).

A third protocol imitated key steps of hippocampal development and generated a few *NeuroD1*-, *Prox1*- and *Tbr1*-positive granule-like cells. In this protocol, hESC-derived EB were first treated with the anti-caudalizing proteins DKK1 (WNT antagonist), NOGGIN (BMP-antagonist), the ALK5 (TGFB pathway) inhibitor SB431542 and the SHH inhibitor cyclopamine. Then, WNT3a and brain-derived neurotrophic factor (BDNF) were added and co-cultures were initiated as single cell suspensions on hippocampal astrocytes. The resultant DG precursor-like cells were then transplanted into the hippocampus of brains *in vivo*, and their integration and maturation were followed. However, this study lacks detailed co-localization studies with DG markers, so that the final differentiation phenotypes of the DG-like cells cannot be assessed (we do not know if these cells are really granule cells; Yu et al., 2014a). Furthermore, the last stages of differentiation with this protocol could be achieved only after *in vivo* transplantation. This indicates that the *in vitro* differentiation protocols are missing key factors present in the *in vivo* local stem cell niche, and also argues for a closer look at these niches and their molecular mechanisms. In the same direction, it is intriguing that hiPSCs can differentiate into *Prox1*-positive granule-like cells with typical DG granule cell dendrites simply by co-culture with hippocampal slice cultures (Hiragi et al., 2017).

Interestingly, the differentiation protocol from Yu et al. (2014a) has also been applied to hiPSCs from patients with schizophrenia (Yu et al., 2014a). Here the authors observed lower levels of *NeuroD1*, *Prox1* and *Tbr1* expression as well as reduced neuronal activity and spontaneous neurotransmitter release.

## Electrophysiological Features of Immature and Mature Granule Cells

Immature newborn granule cells play key roles in learning and memory because of their augmented synaptic plasticity and excitability (Kempermann et al., 1997; Cameron and McKay, 2001; Ninkovic et al., 2007); on the contrary, mature granule cells are reported to be more resistant to excitatory inputs (Liu et al., 2012; Ramirez et al., 2013; Redondo et al., 2014; Ryan et al., 2015). After generating DG granule like-neurons *in vitro* from iPSCs it is important to determine if they show the electrophysiological features of either immature or mature *in vivo* DG granule cells.

Differential parameters between both kinds of neurons include intrinsic properties—input resistance (Rin), capacitance and action potential waveform—as well as synaptic properties (esp. spontaneous postsynaptic potentials/currents).

One key neuronal parameter, the resting membrane potential (Vm), could in principle be used to assess the functional status of neurons. However, the Vm of immature DG granule cells has been variously reported in the literature. Among others found relatively depolarized Vm values (around −50 mV) in immature DG granule cells, and (Pedroni et al., 2014) even report −30 to −40 mV. Careful measurements by other researchers however have yielded higher values, similar to those found for mature DG granule neurons (around −80 mV; Schmidt-Hieber et al., 2004). Furthermore, (Mongiat et al., 2009) found −75 mV for immature and −80 mV for mature, and numerous other authors have recorded Vm values for immature DG granule cells similar to those of mature ones (Ge et al., 2006; Heigele et al., 2016; Li et al., 2017). These differences depend probably on the technical difficulties involved in performing patch clamp recordings on cells with very high Rin. This has been discussed in detail by Heigele and collaborators (see the “Methods” section in Heigele et al., 2016).

More agreement is found around the differential character of the Rin, which is remarkably larger for immature than mature DG granule cells. The simpler dendritic morphology, shorter axonal processes and lower expression of ion channels of immature cells enhance their Rin significantly, often up to GΩ (Schmidt-Hieber et al., 2004; Mongiat et al., 2009; Pedroni et al., 2014).

The membrane capacitance of immature DG granule cells has also been consistently found smaller than that of mature ones. The actual values and the degree of difference vary between reports. For instance, (Heigele et al., 2016) have reported 19.2 ± 1.5 vs. 69 ± 5 pF, while (Pedroni et al., 2014) found 14 ± 1 vs. 17 ± 1 pF, and (Mongiat et al., 2009) obtained 30.6 ± 1 vs. 56.9 ± 2.1 pF.

Perhaps the best approach would be to base the assessment of maturity on Rin and capacitance, instead of Vm, which is more difficult to assess correctly on immature DG granule cells.

The waveform of the action potential becomes more narrow with maturation, probably because of a decrease of calcium-spikes with superimposed action potentials. In contrast, mature adult granule cells are reported to be less excitable and their sparse firing *in vivo* is well known (Liu et al., 2012; Ramirez et al., 2013; Redondo et al., 2014; Ryan et al., 2015). In addition, the network activity of most of immature spiking neurons is strongly synchronized, resembling the typical giant depolarizing potentials (GDPs) of the hippocampal formation. These GDPs are generated by the synergistic actions of glutamate and GABA which are, at this immature stage, both depolarizing and excitatory (Pedroni et al., 2014).

Immature DG granule cells show a clear GABA ergic, rather than glutamatergic, phenotype (Schmidt-Hieber et al., 2004; Esposito A. et al., 2005; Pedroni et al., 2014). Transient production of GABA (expression of glutamic acid decarboxylase) and release of GABA from granule cells during maturation has been observed (Cabezas et al., 2013) and may persist, in attenuated form, into adulthood (Gutiérrez, 2016).

Immature granule cells have another specific characteristic: under physiological conditions, the transient T-type calcium conductance expression underlies a low-threshold calcium spike, which again greatly enhances the initiation of superimposed sodium spikes (Schmidt-Hieber et al., 2004). On the contrary, mature granule cells fail to produce low-threshold calcium spikes. Under pharmacologically blocked T-type channels, no significant modification could be observed in their sodium spike threshold (Schmidt-Hieber et al., 2004; Martinello et al., 2015). Mature granule cells exhibit low-threshold calcium spikes when potassium channel blockers are present (Blaxter et al., 1989), and are capable of firing action potential exacerbations under physiological conditions, unlike immature granule cells (Staley et al., 1992; Pernía-Andrade and Jonas, 2014). This fact increases the opportunities for triggering spikes in their CA3 pyramid targets (Henze et al., 2002).

In the dendrites of both mature and immature granule cells, sodium action potentials cause calcium transients with high amplitude (Schmidt-Hieber et al., 2007; Stocca et al., 2008; Hamilton et al., 2010; Krueppel et al., 2011; Kamijo et al., 2014). Yet, a more efficient transient input summation is made possible by immature granule cells, which exhibit longer transients in proximal and distal dendrites. This fact appears to be caused by lower endogenous calcium binding capacities and slower extrusion rates (Stocca et al., 2008). For mature and immature cells, variations in the calcium influx could basically enhance distinctions in synaptic plasticity.

Mature granule cell dendrites can be considered passive linear integrators with a quite intense voltage signal attenuation (Krueppel et al., 2011). In general, mature granule cells have electrophysiological features characterized by normal action potential amplitude and sparse firing (Mongiat et al., 2009), strong perisomatic GABAergic inhibition (Cupello et al., 2005; Esposito A. et al., 2005; Temprana et al., 2015), efficient calcium buffering (Stocca et al., 2008) and a high threshold for long term potentiation induction (Wang et al., 2000; Schmidt-Hieber et al., 2004; Ge et al., 2007).

Finally, let us remark that the degree of maturation of DG granule cells generated *in vitro* can be enhanced by co-culture with hippocampal astrocytes and/or neurons, or by culturing them on the GCL of hippocampal organotypical slices.

## Granule Neurons of the DG: Pathologies and Models

### Granule Neurons of the DG in Disease

A number of neuropathological defects causing higher cognitive dysfunction are concomitant with abnormal hippocampal neurogenesis and plasticity (Xu C. J. et al., 2015). In newborn granule neurons, neurogenesis and development of irregular hilar basal dendrites are influenced by hippocampal epileptic seizures (Jessberger et al., 2007; Hattiangady and Shetty, 2010). Regular granule neurons develop dendrites towards the molecular layer and send axons through the hilus. On the contrary, dendrites from neurons that are induced by seizures appear to grow exuberantly into the hippocampal hilus instead of the molecular layer.

In the aging brain, the decrease in hippocampal neurogenesis is due to the declining neural progenitor pool (Jessberger and Gage, 2008; Villeda et al., 2011; Spalding et al., 2013). It has been documented that deficiencies in the hippocampus are related to specific cognitive disorders: depression (Patrício et al., 2013), early stages of Alzheimer’s disease (Demars et al., 2010; Faure et al., 2011; Mu and Gage, 2011; Rodríguez et al., 2011), bipolar disorder (Valvezan and Klein, 2012; Walton et al., 2012) and schizophrenia (Tamminga et al., 2010; Walton et al., 2012; Hagihara et al., 2013). Moreover, there is reason to think that the etiology and pathogenesis of schizophrenia and bipolar disorder are related to abnormal DG granule neuron maturation (Reif et al., 2006; Yamasaki et al., 2008; Walton et al., 2012; Hagihara et al., 2013; Shin et al., 2013).

NSC research has advanced very fast in the last few years, in particular in the fields of *in vitro* development, cellular reprogramming, cell differentiation and regenerative therapeutic techniques such as cell transplantation. Analyzing the pathways used by pluripotential stem cells (PSCs) to differentiate into particular neuronal and glial phenotypes has extended our knowledge about human brain development and maturation and will help in the future to understand certain neurological disorders. The generation of specific neuronal cell types and brain structures *ex vivo* will be an additional important step in brain research, to regulate the directed neuronal differentiation of crucial brain development events in stem cells and neuronal precursors.

For this reason, an exceptional opportunity for modeling neurological disorders and for drug design may be provided by the generation of DG granule neurons *in vitro* from the cells of patients with hippocampal CNS disorders.

### Induced PSCs and Models

Harvesting central nervous system (CNS) tissue from living patients suffering brain disorders it is not possible. By using patient-derived neural induced PSCs (iPSCs), analysis of CNS disorders is now within reach of scientists and clinicians. Neural iPSCs are able to form functional neurons and glial cells; however, developing differentiation strategies for the generation of specific neurons and glia cells and their disease-relevant subtypes is still a significant challenge. A series of breakthroughs in the field of neural human PSCs (hPSC) differentiation during the past decade, has made it possible to use iPSCs as the basis of CNS disease models (for reviews see Sandoe and Eggan, 2013; Okano and Yamanaka, 2014; Pasca et al., 2014; Srikanth and Young-Pearse, 2014). iPSC technology has already been used to generate ventral midbrain dopaminergic neurons for Parkinson disease (Perrier et al., 2004; Roy et al., 2006; Kriks et al., 2011; Ma et al., 2011; Kikuchi et al., 2017), spinal motor neurons for amyotrophic lateral sclerosis (Di Giorgio et al., 2008; Dimos et al., 2008; Marchetto et al., 2008), cortical pyramidal neurons (Shi et al., 2012; Vanderhaeghen, 2012) and forebrain interneurons (Maroof et al., 2013; Nicholas et al., 2013; Yuan et al., 2015; Sun et al., 2016).

Furthermore, it has been recently shown that so-called cerebral organoids can be generated *in vitro* from iPSCs. These are 3D-cultured cells that self-assemble into brain-like structures reproducing aspects of cerebral developmental and even showing discrete regional cortical organization (Lancaster et al., 2013; Pasca et al., 2015; Renner et al., 2017; Sloan et al., 2017). Appropriately-arranged germinal zones and radial and tangential migration of cortical neurons can be seen in these organoids as well as cortical organizers such as the CH (Renner et al., 2017). This suggests that the organoids generate neuronal micro-environments and are self-organizing. In the present stage, the generation of dorsal forebrain structures like the hippocampus by organoids succeeds only to a limited degree. However, it presents an interesting starting point for tissue-engineering of the simplest cortical structures, including the DG.

The laboratories of Sasai and Gage were the first to obtain hippocampal and DG-like neurons from embryonic stem cells (ESCs; Watanabe et al., 2005; Yu et al., 2014a; Sakaguchi et al., 2015). In one of these studies, researchers applied a directed differentiation protocol to generate human granule-like cells by reprogramming cells from patients with schizophrenia (Yu et al., 2014a). In principle, *in vivo* cell therapies could be developed on the basis of this kind of patient-specific, individualized generation of DG neurons from hiPSCs. Obviously, there remains a lot of extra work to make cell transplantation into a practicable therapy. Despite these difficulties, numerous studies have applied stem cell derived neuronal transplantation successfully in animal models and achieved functional improvements. These early successes emphasize the need for further research in order to understand the exact mechanisms of function recovery after cell transplantation. This is a vital step for developing effective and safe *in vivo* cell therapies for human patients (Lu et al., 2003; Valente et al., 2006; Wernig et al., 2008; Hargus et al., 2010; Jiang et al., 2011; Ma et al., 2013).

## Outlook and Critical Aspects

The ability to reconstruct the DG *in vitro* would be a major step for examining the molecular and cellular mechanisms underlying neurological disorders (such as schizophrenia, Alzheimer’s disease and temporal lobe epilepsy) as well as for the screening of appropriate therapeutic drugs. The immediate goal to this end is to develop and refine protocols for the reliable production of DG granule cells from ESC- or iPSC-derived cells. The best existing method for the generation of DG granule cells *in vitro* with EB and single cell culturing is still very inefficient (Sakaguchi et al., 2015).

Dorsal telencephalic precursor cells, generated *in vitro* and identified by co-expression of *forkhead box G1 (Foxg1), Gli3, Emx2* and* Ascl1* should provide the starting cell source for the directed differentiation of DG granule cells. Guiding the differentiation of neurons with dorsal telencephalic phenotype through various embryonic stages towards the DG granule neuron phenotype will require a strict following of the key developmental events, starting with the sequential administration of appropriate growth factors and transcription factors, mimicking the differentiation of the DG neuroepithelium towards the different proliferation matrices and finally immature and mature granule cells. Major immediate challenges concern the scheduling and titration of these factors. In this context, it would be most interesting to analyze possible synergistic effects of BMP7 and WNT3a on the production of *Emx2/Lef1*-expressing IPCs and of the *Prox1/NeuroD1/Calb2*-expressing neurons that they generate during DG granule cell differentiation *in vitro*. IPC differentiation into DG granule cells is enhanced by expression of key transcription factors, particularly *Emx2*, *Neurog2*, *Tbr2*, *NeuroD1*, *Lef1* and *Prox1* (Hodge et al., 2008, 2012).

DG granule cell differentiation could also be forced by sequential and coordinated expression of the essential transcription factors. Obviously, when attempting to differentiate neuronal stem cells into granule cells *in vitro*, expression of the key dorsal telencephalic transcription factors needs to be activated in a sequence and time course analogous to those that occur during normal brain development (Li and Pleasure, 2005, 2007). Possible sources of error when assessing the results of protocols for *in vitro* production of DG granule cells are other neurons expressing similar combinations of transcription factors. For instance, the combination *Emx2*-*Tbr2*-*NeuroD1*-*Lef1* is also expressed by pyramidal cells of the CA, and *Emx2*-*Tbr2* are also expressed by CR cells. *Ccnd2* and transcription factors like *Nfix*, *Tlx* and *Ascl1*, which play a major role in postnatal and adult granule cell neurogenesis, will have to be tested for their influence on the proliferation and maintenance of already generated granule cell precursors as well as differentiated granule cells.

Ultimately, single cell expression profiling of cells marked with stage-specific reporter constructs could be needed in order to molecularly define with precision the differentiation stages of granule cells *in vivo* and *in vitro*. Subsequently, conditional overexpression of transcription factors during the directed differentiation of PSCs towards DG granule precursor could be attempted. Lineage tracing of the different stages of granule cell development in combination with single cell expression profiling would be of importance to analyze the sequence of molecular events determining granule cell fate.

Alternatively, the cellular engineering of organizers such as the CH could be attempted. However, the 3-D cultivation of neuronal tissues *in vitro* is a major challenge, particularly for complex regions of the nervous system (Watanabe et al., 2005; Yu et al., 2014a; Sakaguchi et al., 2015; Renner et al., 2017). The ultimate goal will be the generation of 3-D hippocampal organoids (Eiraku et al., 2008; Nakano et al., 2012; Kadoshima et al., 2013; Lancaster et al., 2013; Sasai, 2013a, b; Renner et al., 2017) or at least the reproduction of hippocampal microcircuits on micro-patterned fluidic plates.

Finally, further characterization of the molecular guidance cues of DG granule cells, *in vivo* and *in vitro*, is essential to elaborate protocols generating viable, transplantable neuronal cells; consider for instance the problems posed by the axonal growth-inhibiting milieu of the adult nervous systems (Skutella and Nitsch, 2001; Ng et al., 2013; Senzai and Buzsáki, 2017). When performing neuronal cell therapy after brain traumata or neurodegenerative disorders, it is crucial to deliver the transplanted cells to the exact location of damage and to integrate them physiologically and functionally. For the generation of appropriate neuronal connections, the transplanted cells have to develop dendrites and axons able to find their appropriate functional partners and interact with them. At the end of the process, the transplanted cells need to be functionally integrated into the respective neuronal circuit.

Growth-inhibition factors for re-growing axons and dendrites in the adult CNS post-lesion make this integration process difficult. Therefore, it is essential to support the outgrowth and navigation of neuronal processes and their integration in the existing entorhinal-DG-CA circuitry. This can be attempted by administering anti-myelin-associated factors (Xu L. et al., 2015). The functional implications of the three myelin-derived inhibitors NogoA (reticulon 4, Rtn4), myelin-associated glycoprotein and oligodendrocyte-myelin glycoprotein as well as a number of axon guidance molecules that control the survival, proliferation, migration and differentiation of NSCs has been reviewed recently (Song et al., 2018).

Despite of all accomplishments in this field, a long way is still ahead for the generation of *in vitro* differentiated neurons identical to their natural models.

## Author Contributions

MH and SC drafted the article. GA-B and TS wrote the final version. PN drew the Figure.

## Conflict of Interest Statement

The authors declare that the research was conducted in the absence of any commercial or financial relationships that could be construed as a potential conflict of interest.

## Abbreviations

1stM: First Matrix
2ndM: Second Matrix
3rdM: Third Matrix
Acvr1: activin receptor type 1 (gene)
Apc: adenomatosis polyposis coli (gene)
Apcdd1: APC down-regulated 1 (gene)
Ascl1: achaete-scute family bHLH transcription factor 1 (gene)
Bdnf: brain-derived neurotrophic factor (gene)
Bmpr1a: bone morphogenetic protein receptor 1a (gene)
BMPs: bone morphogenetic proteins
CA: Ammon’s horn (Cornu Ammonis) of the hippocampus
Calb1: calbindin 1 (gene)
CcnD2: cyclinD2
CDKs: cyclin-dependent kinases
CH: cortical hem
ChP: Choroid Plexus
CNS: central nervous system
CP: Choroid Plaque
CR: Cajal-Retzius cells
Cxcr4: chemokine receptor type 4
DG: dentate gyrus
Dkk: Dickkopf-related protein
DNE: Dentate Neuroepitelium
EB: embryoid body
Egf: epidermal growth factor (gene)
Emx1: empty spiracles homebox 1 (gene)
Emx2: empty spiracles homebox 2 (gene)
F: fimbria
FGF: fibroblast growth factor
Foxg1: forkhead box G1 (gene)
GABA: gamma-aminobutyric acid
GDFs: growth and differentiation factors
GDPs: giant depolarizing potentials
GFs: growth factors
Gli3: Glioma-Associated Oncogene Family Zinc Finger 3 (gene)
GRIK4: glutamate receptor, ionotropic, kainate 4 (human gene)
KA1: GΩ, giga Ohms
Hes5: hairy and enhancer of split 5 (gene)
HF: hippocampal fissure
HNE: Hippocampus Neuroepithelium
hPSC: human PSCs
Id: inhibitor of DNA binding
IPCs: intermediate precursor cells
iPSCs: induced PSCs
KA1: GRIK4 (gene)
LacZ: β-galactosidase
Lef1: lymphoid enhancer binding factor 1
Lhx5: LIM homeobox protein 5
Lrp6: lipoprotein receptor-related protein 6
MS: Migratory Stream
mV: millivolts
NeuN: “Neuronal Nuclei”, neuron-specific nuclear antigen, mouse nuclear protein Rbfox3, RNA binding protein, fox-1 homolog (*C. elegans*, specific neuronal marker)
NeuroD1: neuronal differentiation 1 (gene)
Neurog2: neurogenin 2 (gene)
Nfix: nuclear factor I/X (gene)
Nr2e1: nuclear receptor subfamily 2, group E, member 1 (gene)
NSCs: neural stem cell
Pax6: paired box 6 (gene)
pF: picofarad
Prox1: prospero homeobox 1 (gene)
PSCs: pluripotent stem cells
Ptch1: receptor patched 1 (gene)
Rin: input resistance
Rspo: R-spondins (gene)
Sdf1: stromal cell-derived factor 1 (gene)
Sfrp2: secreted frizzled-related protein 2 (gene)
Shh: sonic hedgehog (gene)
Smo: smoothened (gene)
Tbr2: T-box brain protein 2 (gene)
TCF/LEF: transcription factor/lymphoid enhancer binding factor, a family of transcription factors in the Wnt pathway
TF: transcription factor
TGFB: transforming growth factor beta, signaling protein with four isoforms (1–4)
TGFB1: transforming growth factor beta 1 (gene)
Vm: membrane resting potential of a neuron
VZ: ventricular zone
WNT: Wnt signaling pathway
ZBTB20: Zinc finger and BTB domain-containing protein 20 (gene).
